# Curcumin against gastrointestinal cancer: A review of the pharmacological mechanisms underlying its antitumor activity

**DOI:** 10.3389/fphar.2022.990475

**Published:** 2022-09-02

**Authors:** Yuanyuan Fan, Xiqin Zhang, Yuxin Tong, Suning Chen, Jingjing Liang

**Affiliations:** ^1^ Department of Traditional Chinese Medicine, Shengjing Hospital of China Medical University, Shenyang, China; ^2^ Liaoning Key Laboratory of Research and Application of Animal Models for Environmental and Metabolic Diseases, Medical Research Center, Shengjing Hospital of China Medical University, Shenyang, China

**Keywords:** gastrointestinal cancer, gastric cancer, colorectal cancer, curcumin, pharmacological mechanism

## Abstract

Gastrointestinal cancer (GIC) poses a serious threat to human health globally. Curcumin (CUR), a hydrophobic polyphenol extracted from the rhizome of *Curcuma longa*, has shown reliable anticancer function and low toxicity, thereby offering broad research prospects. Numerous studies have demonstrated the pharmacological mechanisms underlying the effectiveness of CUR against GIC, including the induction of apoptosis and autophagy, arrest of the cell cycle, inhibition of the epithelial–mesenchymal transition (EMT) processes, inhibition of cell invasion and migration, regulation of multiple signaling pathways, sensitization to chemotherapy and reversal of resistance to such treatments, and regulation of the tumor survival environment. It has been confirmed that CUR exerts its antitumor effects on GIC through these mechanisms *in vitro* and *in vivo*. Moreover, treatment with CUR is safe and tolerable. Newly discovered types of regulated cell death (RCD), such as pyroptosis, necroptosis, and ferroptosis, may provide a new direction for research on the efficacy of CUR against GIC. In this review, we discuss the recently found pharmacological mechanisms underlying the effects of CUR against GIC (gastric and colorectal cancers). The objective is to provide a reference for further research on treatments against GIC.

## 1 Introduction

Gastrointestinal cancer (GIC) is characterized by high incidence and mortality rates, posing a serious threat to human health globally. According to the Global Cancer Statistics 2020 estimates (Sung et al., 2021), gastric cancer (GC) ranked fifth and fourth among cancers in terms of incidence (>1 million new cases) and mortality (769,000 deaths), respectively. Similarly, colorectal cancer (CRC) accounts for >1.9 million new cases and 935,000 deaths, ranking third and second among cancers, respectively.

Surgical treatment, radiotherapy, and chemotherapy have traditionally been the main strategies for the treatment of GIC. However, owing to the low rate of early detection and high rate of postoperative recurrence associated with GIC, the effectiveness of radiotherapy and chemotherapy may be compromised by the occurrence of severe undesirable side effects. In the search for effective treatments with fewer side effects, an increasing number of research studies are focused on traditional herbal medicines and their monomer compounds.

Curcumin (CUR) was first discovered by Vogel and Pelletier (Prasad et al., 2014), and is the most important component of the rhizomes of turmeric (Curcuma longa) (Waly et al., 2018). CUR is a hydrophobic polyphenol that has been approved by the US Food and Drug Administration based on its bio-safety (Mashayekhi-Sardoo et al., 2021). Moreover, it has demonstrated a wide range of pharmacological activities, such as antibacterial (Ibarra-Martinez et al., 2022), anti-inflammatory (Yan et al., 2021), antioxidant (Xu et al., 2021), and antitumor (Zhang et al., 2020). CUR has been widely reported to inhibit the proliferation of tumor cells in a concentration-and time-dependent manner *in vitro* (Li et al., 2017a; Fan et al., 2020; Mao et al., 2021). According to relevant clinical trials on safety and toxicity, the acceptable dose of CUR for maximum efficacy is 4–8 g per day. It has been reported that humans can tolerate treatment with CUR at a dose up to 12 g per day (Barati et al., 2019).

**FIGURE 1:**
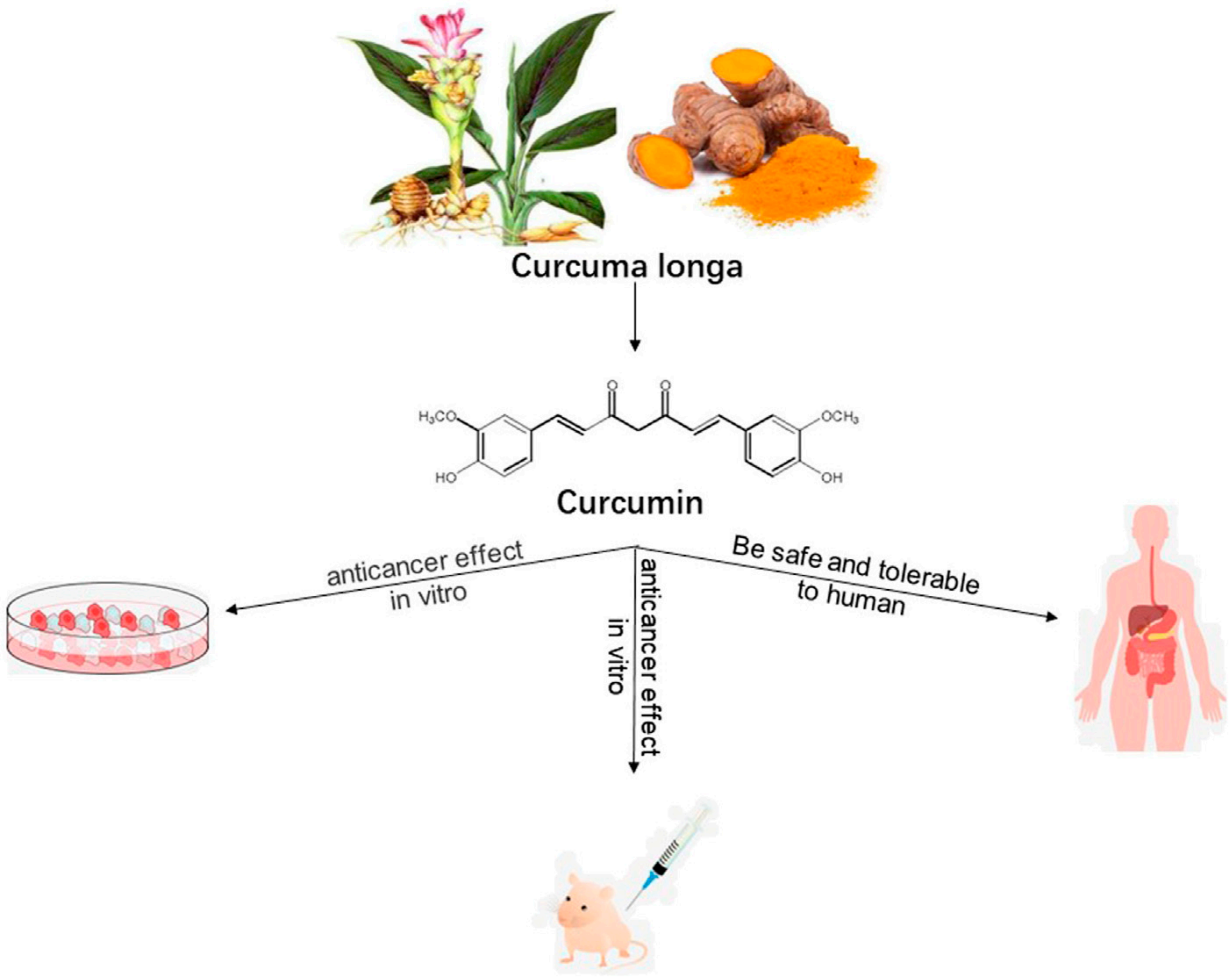
Research method utilized to summarize the pharmacological action of curcumin against gastrointestinal cancers.

In this review article, we discuss the pharmacological mechanisms underlying the effects of CUR against GIC based on recent evidence derived from *in vitro* and *in vivo* experiments, as well as clinical trials (Figure 1). The objective of this review is to provide a reference for further research on the treatment of GIC using CUR.

## 2 Curcumin in gastrointestinal cancer (gastric cancer and colorectal cancer)

### 2.1 Inhibition of gastrointestinal cancer cell proliferation and blockage of cell cycle

It is widely recognized that CUR inhibits the proliferation and cell cycle of GIC cells. Cyclin D1 (CCND1) and cyclin-dependent kinase 4 (CDK4), a serine/threonine kinase which binds to CCND, can regulate cell transition from G1 to S phase (Yin et al., 2018). Sun et al. (Sun et al., 2019) found that CUR significantly inhibited cell cycle progression of SGC7901 cells in the G0/G1-S phase, increased the cell number in the G0/G1 phase, and downregulated the expression of B-cell lymphoma 2 (Bcl-2), CDK4, and CCND1 proteins in both cells and tissues.

Herrero et al. (De La Parte et al., 2021) found that CUR inhibited the proliferation of CRC cells CC531 *in vitro* and reduced the tumor volume in liver implants *in vivo*. Mao et al. reported that CUR inhibited tumorsphere formation and reduced cell viability in a concentration-dependent manner in LGR5 (+) colorectal cancer stem cells (CSCs). Inhibition of the proliferation of HCT-116 and LoVo CRC cell lines by CUR has also been reported (Mao et al., 2021).

Blakemore et al. (Blakemore et al., 2013) elucidated the stage of arrest induced by CUR in eight CRC cell lines (i.e., Caco-2, DLD-1, HCA-7, HCT116p53+/+,HCT116p53^−/−^, HCT116p21^−/−^, HT-29, and SW480). The results showed that the majority of these eight cell lines were arrested at the G2/M transition, with some cells arrested in mitosis. Notably, HCT116 and Caco-2 cells presented the highest levels of mitotic arrest.

### 2.2 Induction of cell apoptosis

Apoptosis is an indispensable mechanism for maintaining normal and healthy bodily functions. Any change in apoptotic patterns can enhance the survival and progression of cancer cells (Li et al., 2017b; Liang et al., 2018). CUR can promote the apoptosis of tumor cells by regulating multiple signaling pathways, upregulating proapoptotic genes, or downregulating antiapoptotic genes, thus inhibiting cancer progression (Liang et al., 2018).

#### 2.2.1 Activation of the p53 signaling pathway

The tumor suppressor gene p53 plays an important role in the pathology of human cancer. The protein p53 inhibits the growth of cancer cells by regulating cell cycle arrest, cell apoptosis, and DNA repair (Hafner et al., 2019). Upregulation of p53 expression inhibits the proliferation and induces apoptosis of cancer cells (Su et al., 2017).

It has been shown that treatment with CUR for 24 h inhibited the proliferation and apoptosis of GC cells SGC-7901 and BGC-823 in a dose-dependent manner. Further studies found that this effect was related to the activation of the p53 signaling pathway through the upregulation of p53 and p21 (Fu et al., 2018).

By taking advantage of the waiting period before surgery, He et al. (2011) treated a group of CRC patients with CUR. The results showed that weight loss was improved in patients treated with CUR. In addition, by analyzing in human CRC tissue specimens obtained at the time of diagnosis (biopsy via colonoscopy) and surgery (surgical removal of CRC tissue), they demonstrated that CUR reduced the serum levels of TNF-α, induced apoptosis of CRC cells, upregulated p53 expression, and regulated the expression of apoptosis-related Bax and Bcl-2.

#### 2.2.2 Inhibition of the phosphoinositide-3-kinase/Akt (PI3K/Akt) signaling pathway

The PI3K/Akt/mechanistic target of rapamycin kinase (PI3K/Akt/mTOR) signaling pathway is a well-studied antiapoptotic and pro-proliferative signaling pathway (Braglia et al., 2020). It has been revealed that this pathway is activated in approximately one-third of human cancers (Khorasani et al., 2021). Abnormal activation and overexpression of key signal molecules in the PI3K/Akt signaling pathway may promote the occurrence and development of GIC.

By treating SGC-7901, BGC-823, and MKN28 cell lines with increasing amounts of CUR for 24, 48, and 72 h, Li et al. (2017a) found that CUR could inhibit the proliferation and induce apoptosis of GC cells in a time- and dose-dependent manner. The results of western blotting assays showed that treatment with CUR downregulated the expression of phospho-Akt together with that of downstream phospho-mTOR and phospho-p70s6K in a dose-dependent manner. These findings indicated that the activation of the PI3K/Akt/mTOR signaling pathway was suppressed in GC cells following treatment with CUR. Liu et al. (2018) reported that CUR could inhibit the PI3K/Akt signaling pathway and activate the activity of p21 protein, thus promoting apoptosis in GC cell line AGS.

It has been reported that CUR downregulated PI3K and Akt expression while promoting apoptosis in the early stages of CRC induced by 1,2-dimethylhydrazine dihydrochloride in rats (Rana et al., 2015). Also, the results of western blotting assays showed that CUR upregulated the expression of pro-apoptotic Bcl-2 family members (Bcl-2 associated agonist of cell death [Bad] and Bax) and cysteine protease family apoptosis executioner (CASP3/9), whereas it downregulated that of antiapoptotic Bcl-2 protein.

#### 2.2.3 Inhibition of the Wnt/β-catenin signaling pathway

β-catenin can be activated by Wnt signaling and is involved in different types of signal transduction. Studies have confirmed that it is essential for tumor growth (Zheng et al., 2017). The regulation of the Wnt/β-catenin signaling pathway is highly relevant to the metabolism of cancer cells (Cantor & Sabatini, 2012), and plays an important role in the occurrence, progression, and prognosis of various types of cancer in humans. Activation of the Wnt/β-catenin signaling pathway results in the development of resistance to chemotherapy in several types of cancer (Sun et al., 2012; Nagaraj et al., 2015). Therefore, it is hypothesized that it could effectively inhibit tumor growth by targeting the Wnt/β-catenin signaling pathway (Zheng et al., 2017).

Zheng et al. (2017) found that CUR significantly attenuated cell viability and induced tumor cell apoptosis *in vitro* in GC cell lines SNU-1, SNU-5, and AGS. CUR significantly suppressed the levels of Wnt3a, LDL receptor related protein 6 (LRP6), phospho-LRP6, β-catenin, phospho-β-catenin, c-Myc, and survivin, demonstrating that Wnt/β-catenin signaling was downregulated. In an *in vivo* experiment, AGS cells were used to establish a xenograft tumor model in male BALB/c nude mice. The CUR treatment group received CUR (1 mg/kg body weight) through gavage once daily, and the results showed that xenograft growth was inhibited by treatment with CUR.

The Wnt/β-catenin signaling pathway has been considered one of the most frequently dysregulated pathways in CRC (McCubrey et al., 2016). Its abnormal activation is related to cell proliferation, invasiveness, and resistance to therapy, indicating its potential value as a target in the treatment of CRC (Bahrami et al., 2017). Li et al. (2018) found that high levels of paternally expressed gene 10 (PEG10) increased the expression of Wnt1 and β-catenin in colon tissues, and this effect may be regulated by miR-491. By upregulating miR-491 and inhibiting the Wnt/β-catenin signaling pathway, CUR inhibited the expression of PEG10, thereby inducing apoptosis and impairing the proliferation of HCT-116 cells.

Axin2, a downstream gene of the Wnt/β-catenin signaling pathway, has been associated with cell proliferation, mutation, migration, and apoptosis (Gunes et al., 2010; Aristizabal-Pachon et al., 2015). Hao et al. (Hao et al., 2021) found that the Wnt/β-catenin pathway was activated in human CRC samples, accompanied by an increased mRNA expression of Axin2. Furthermore, they demonstrated that CUR could reduce Axin2 in the Wnt/β-catenin pathway, thus inhibiting CRC *in vivo*.

#### 2.2.4 Induction of reactive oxygen species production

Reactive oxygen species (ROS), a general term describing oxygen or oxygen-containing highly reactive molecules, acts as a double-edged sword for cancer cells; these molecules act differently at different stages of cancer (Prasad et al., 2017). ROS can promote the proliferation and growth of cancer cells by activating a variety of cell signaling pathways, while the excessive accumulation of ROS can lead to cell damage and apoptosis (Liang et al., 2021).

Tong et al. (Tong et al., 2020) found that high concentrations of CUR (≥20 μmol/L) increased the levels of ROS in human GC cells, leading to mitochondrial damage, DNA damage, and apoptosis. These findings indicated that the anticancer effect of CUR in GC may be closely related to its pro-oxidative effect at high concentrations and elevation in the levels of ROS in cancer cells. In addition, the investigators demonstrated that CUR induced DNA demethylation in human GC cells through the damaged DNA repair-p53-p21/growth arrest and DNA damage inducible alpha-cyclin (GADD45A-cyclin)/CDK-retinoblastoma/E2F-DNA methyltransferase1 (E2F-DNMT1) axis.

Liang et al. (2014) found that CUR induced apoptosis combined with the production of ROS in BGC-823 cells. The apoptosis induced by CUR was reduced by adding an antioxidant (N-acetyl cysteine [NAC] or trion) to inhibit ROS production. Additional experiments showed that the apoptotic signal regulating the kinase 1-mitogen-activated protein kinase 4-JUN N-terminal kinase (ASK1-MKK4-JNK) signaling pathway was activated by oxidative stress induced by CUR, further leading to apoptosis.

Sritharan & Sivalingam (2021) found that treatment with CUR induced apoptosis, ROS generation, and a decrease in MMP in HT-29 and HCT-116 cell lines. Pre-treatment with NAC effectively reduced the levels of ROS and prevented MMP loss in HT-29 cells; however, it was less effective in HCT-116 cells. Pre-treatment of HCT-116 cells with NAC for 24 h upregulated the protein expression of total p53. This evidence demonstrated that CUR induced ROS-mediated apoptosis in CRC cell lines, and this effect may be mediated by p53.

#### 2.2.5 Activation of the mitochondrial pathway

Liu et al. (2014) treated SGC7901 cells with co-administration of CUR at serial concentrations and diazoxide–an adenosine triphosphate-sensitive (ATP-sensitive) potassium (KATP) channel opener. They found that CUR induced the opening of KATP channels, leading to loss of the mitochondrial membrane potential (MMP) in a dose-dependent manner; moreover, it induced apoptosis of tumor cells.

Inhibition of glycolysis has been a therapeutic target in cancer (Hamanaka & Chandel, 2012). The regulation of key glycolytic enzymes, such as hexokinase 2 (HK2), is an effective approach to the inhibition of glycolysis (Patra et al., 2013). Wang K. et al (2015) found that the uptake of glucose, as well as the production of ATP and lactic acid, were inhibited by CUR in a dose-dependent manner in human CRC cells HCT116 and HT29. Further study showed that CUR induced the dissociation of HK2 from the mitochondria and triggered mitochondria-mediated apoptosis. Collectively, the evidence demonstrated that CUR inhibited glycolysis and promoted mitochondria-mediated apoptosis by regulating HK2 in CRC cells.

Similarly, it has been reported that CUR induced apoptosis through the mitochondrial cell death pathway in LoVo cells (Guo et al., 2013). Guo et al. found that CUR induced apoptosis in LoVo cells, accompanied by the release of lactate dehydrogenase, collapse of MMP, arrest of the cell cycle, and activation of CASP3/9. Moreover, the results of western blotting analysis showed that CUR upregulated the expression of cytochrome c, Bax, and p53, whereas it downregulated that of Bcl-2 and survivin in LoVo cells.

### 2.3 Induction of autophagy

Autophagy is a catabolic degradation process in which cellular proteins or organelles are degraded in the lysosome and recycled. This process plays an important and complicated role in the occurrence and development of cancer, as well as the development of resistance to therapy (Li et al., 2017a). Studies utilizing transmission electron microscopy revealed the presence of autophagosomes in GC cells (SGC-7901 and BGC-823) treated with CUR (Fu et al., 2018). Li et al. (2017a) found that CUR induced the formation of acidic vesicular organelles, conversion of LC3-I to LC3-II, and upregulation of the expression of autophagy-related proteins, such as beclin 1 (BECN1), autophagy related 7 (ATG7), and ATG5–12 conjugate in BGC-823, SGC-7901, and MKN-28 cells. These results demonstrated that CUR induced autophagy in GC cells. Interestingly, CUR and autophagy inhibitor 3-methyladenine co-treatment attenuated the viability of GC cells and increased apoptosis. In conclusion, they suggested that CUR induced a protective autophagy, which could be antagonistic apoptotic cell death in GC cells.

Mao et al. (2021) found that CUR induced autophagy in colorectal CSCs in a concentration-dependent manner. The results obtained from transmission electron micrographs confirmed this finding. In addition, co-treatment with hydroxychloroquine (an autophagy inhibitor) significantly decreased the cell proliferation induced by CUR.

### 2.4 Inhibition of epithelial–mesenchymal transition processes

By converting the epithelial characteristics of cells into mesenchymal attributes, EMT stimulates the progression of cancer. This is achieved by enhancing the invasive, migratory, and metastatic abilities of cancer cells. In recent years, studies have focused on elucidating the mechanism involved in the inhibition of EMT by treatment with CUR in GIC cells (Suarez-Carmona et al., 2017).

#### 2.4.1 Inhibition of the Wnt/β-catenin pathway

The Wnt/β-catenin signaling pathway, an important pathway in EMT, plays an important role in embryogenesis and human diseases, including various types of cancer.

Zhang et al. (2020) reported that CUR inhibited EMT processes, cell migration, invasion, and cytoskeletal remodeling, and induced apoptosis in SGC-7901 cells. The results of reverse transcription-quantitative polymerase chain reaction, western blotting, and co-immunoprecipitation analyses showed downregulation of the mRNA and protein expression of sonic hedgehog signaling molecule (SHH), GLI family zinc finger 1 (GLI1), and forkhead box M1 (FOXM1) in the SHH signaling pathway, and β-catenin in the Wnt signaling pathway. These findings indicated that CUR inhibits the SHH and Wnt signaling pathways. This inhibition of the aforementioned signaling pathway, as well as the addition of CUR, also suppressed the EMT process. In addition, the evidence demonstrated an interaction between GLI1 and β-catenin, which could be inhibited by CUR.

Zhang et al. (2016) found that CUR inhibited EMT in CRC cells via the naked cuticle homolog 2-Wnt-C-X-C motif chemokine receptor 4 (NKD2-Wnt-CXCR4) signaling pathway. The results showed that CUR significantly inhibited the proliferation of CRC cells and upregulated the expression of NKD2 in SW620 CRC cells. These changes resulted in the regulation of key markers (downregulation of β-catenin and transcription factor 4 [TCF4] expression, upregulation of axin) of Wnt signaling. In addition, the progression of EMT was inhibited through the overexpression of E-cadherin and downregulation of vimentin. CUR also inhibited tumor metastasis by significantly reducing the expression of CXCR4.

Chen et al. (2020) reported that CUR downregulated the expression of transcription factors to promote EMT in CRC cells by reducing methylation in the promoter of caudal type homeobox 2 (CDX2) and inhibiting the CDX2/Wnt3a/β-catenin signaling pathway. In human SW480 CRC cells, CUR inhibited EMT, accompanied by the downregulation of DNMT1 and DNMT3a expression, and reduction of the methylation levels in the CDX2 promoter in a concentration-dependent manner. Treatment with CUR reduced the expression levels of N-cadherin, vimentin, Wnt3a, Snail1, and Twist, as well as the nuclear translocation levels of β-catenin in a concentration-dependent manner. In contrast, the expression levels of E-cadherin were increased.

#### 2.4.2 Regulation of miR-200c/EPM5

The tumor suppressor miR-200c is upregulated by CUR in CRC cells (SW620 and HT29) (Wang et al., 2020). As a consequence of EMT repression, cell migration and invasion were also inhibited by CUR. Treatment with CUR also downregulated the expression of EPM5 (also termed PRICKLE2), a direct target of miR-200c. Further investigation indicated that EPM5 acted as a downstream of the CUR/miR-200c cascade, and miR-200c mediated the CUR-repressed EMT by direct repression of EPM5.

### 2.5 Inhibition of invasion and migration

Metastasis of a malignant tumor is often the main reason for failure of therapy. Therefore, an important part of the research on anticancer drugs is focused on the inhibition of tumor metastasis.

Numerous studies (Liu et al., 2018; Sun et al., 2019; Tong et al., 2020; Zhang et al., 2020) have demonstrated that CUR could inhibit the invasion and migration of GC cells. Liu et al. reported that CUR inhibited the expression of miRNA-21 and decreased that of matrix metalloproteinase 2 (MMP2) and MMP9, thus inhibiting the invasion and migration of AGS cells (Liu et al., 2018). Zhang et al. (Zhang et al., 2020) found that CUR affected the migration, invasion, and cytoskeletal remodeling of SGC-7901 cells by regulating the GLI1-β-catenin pathway.

*In vitro* and *in vivo* studies have shown that CUR inhibited the invasion, metastasis, and angiogenesis of CRC cells (Chen et al., 2013; Calibasi-Kocal et al., 2019; De La Parte et al., 2021). According to Chen et al. (Chen et al., 2013), the antimetastatic effect of CUR on CRC cells is related to the downregulation of Sp-1 (transcription factor), FAK (cell adhesion component), and CD24, as well as the upregulation of E-cadherin expression.

Herrero et al. (De La Parte et al., 2021) reported the antitumor effect of CUR against liver implants from CRC both *in vitro* and *in vivo*. Importantly, the investigators quantified the total and individual liver lobe tumor volume in untreated and CUR-treated WAG/RijHsd tumor-bearing rats. They found that CUR reduced the tumor volume in liver implants. Similar findings (Chen et al., 2013) in a mouse model have also been reported.

### 2.6 Sensitization to chemotherapy and reversal of resistance to chemotherapy

#### 2.6.1 Gastric cancer

Chemotherapy, one of the most important treatment options for GC, greatly improves the prognosis and survival of patients with GC. However, the occurrence of chemotherapy-resistant cancer cells has resulted in treatment failure or disease recurrence. Furthermore, the severe adverse effects caused by chemotherapy drugs negatively impact patient quality of life. CUR is a natural plant extract with a good safety profile. Hence, its role in sensitizing cancer cells to chemotherapy and reversing resistance to treatment has become an important topic of research in the GC setting. Drugs commonly used in chemotherapy against GC include 5-fluorouracil (5-FU), cisplatin (DDP), doxorubicin (DOX), etc.

In a study, AGS cells were treated with CUR, DOX, and their combination (DOX-CUR). The results showed that all these treatments significantly inhibited the viability, tumor spheroid formation, migration, and invasion in AGS cells. Notably, the DOX-CUR combination exhibited a more significant anticancer activity than monotherapy with CUR or DOX (Amoodizaj et al., 2020). Yu et al. (2011) found that chemotherapeutics (etoposide and DOX) induced apoptosis and activated NF-κB in SGC-7901 cells. The combination of CUR and chemotherapeutics induced apoptosis, while the activation of NF-κB was attenuated and the expression of the downstream antiapoptotic genes Bcl-2 and Bcl-xL was downregulated.

By establishing a 5-FU-resistant GC cell line (SGCR/5-FU), Kang et al. (2016) found that cytosolic IκBα degradation and NF-κB nuclear translocation were increased in these cells. Treatment with CUR reversed 5-FU resistance and inhibited the proliferation of GC cells by inhibiting the NF-κB signaling pathway. Moreover, downregulation of tumor necrosis factor-α (TNF-α) mRNA was induced by CUR in GC cells.

A regimen combining 5-FU with DDP (FP) is typically used in chemotherapy against GC. He et al. (2017) found that CUR enhanced the effects of treatment with FP on the viability, colony formation, and migratory abilities of MGC-803 cells. This effect may be associated with the apoptosis induced by CUR via the CASP3/8, Bcl-2, and Bax signaling pathways.

The chemotherapeutic regimen combining 5-FU, folinic acid, plus OXA (FOLFOX) plays an important role in the treatment of GC. Nevertheless, it is associated with extensive side effects and the development of resistance in some patients (Kim et al., 2011). Zhou et al. (2016) explored the synergistic antitumor effect of CUR and FOLFOX against GC *in vitro* and *in vivo*. Using cell proliferation assays and assessment of apoptosis, they demonstrated the synergistic antitumor effect of CUR in combination with 5-FU and OXA (5-FU/OXA). This effect may be associated with the downregulation of Bcl-2 expression and upregulation of Bax and CASP3/8/9 expression caused by CUR. In addition, the *in vivo* data showed that the combination of CUR and 5-FU/OXA significantly inhibited the growth of BGC-823 xenograft tumors.

#### 2.6.2 Colorectal cancer

Chemotherapy is an important therapeutic modality in the treatment of CRC. However, toxicity and the development of resistance limit its efficacy. CUR could synergize with DDP to inhibit CRC cells and effectively suppress the occurrence of resistance to DDP in those cells (Zheng Z.-h. et al, 2021; Fan et al., 2022). Fan et al. (2022) reported that this effect of CUR was achieved by targeting the miRNA-137-glutaminase axis. They found that DDP-resistant CRC cells (HT-29) displayed an increased glutamine metabolism, and this elevation could be attenuated by CUR. Further study showed that miR-137, which directly targets glutaminase, was induced by treatment with CUR, leading to sensitization of CRC cells to DDP. According to a study conducted by Zheng et al., the reversal effect of CUR on resistance to DDP in CRC cells was based on the downregulation of lncRNA KCNQ1 opposite strand/antisense transcript 1 (KCNQ1OT1) expression. KCNQ1OT1 is upregulated in HCT8/DDP cells and regulates their proliferation and apoptosis by blocking the suppressive effect of miR-497 on Bcl-2 expression. *In vitro* and *vivo* experiments showed that CUR reversed the development of resistance to DDP in CRC by downregulating KCNQ1OT1 expression, thereby regulating the miR-497/Bcl-2 axis.

It was reported that >15% of patients are resistant to treatment with 5-FU-based chemotherapeutic regimens (Shakibaei et al., 2014). Thus, the resistance to 5-FU is also a research focus in the CRC setting. Studies (Shakibaei et al., 2014; Zhang et al., 2018; Lu et al., 2020; Zheng X. et al, 2021) confirmed that CUR could enhance the sensitivity of CRC cells to 5-FU and reverse the occurrence of resistance. Nicotinamide N-methyltransferase (NNMT) is highly expressed in a wide variety of cancers. It has been reported that NNMT could enhance resistance to 5-FU in human CRC cells (Xie et al., 2016; Li G. et al, 2021). Li et al. (Li G. et al, 2021) found that the effect of CUR on reversing NNMT-induced resistance to 5-FU was related to ROS production and cell cycle arrest. They demonstrated that CUR inhibited the expression of NNMT, though the precise mechanism involved in this process is unclear. Zheng X. et al (2021) showed synergistic inhibitory effects of CUR and 5-FU both *in vivo* and *in vitro* in CRC SW620 cells. These effects may be attributed to inhibition of STAT1 activation to reduce L1 expression and induce a higher level of cell apoptosis. By establishing the 5-FU resistant HCT-116 cell line, Lu et al. (2020) found that CUR reversed resistance to 5-FU in CRC cells through inhibition of the EMT process. This effect may be achieved by regulation of the TET1-NKD-Wnt pathway. Zhang et al. (2018) reported the important role of nuclear factor erythroid 2-related factor (Nrf2) in the effect of CUR on the reversal of resistance to 5-FU in CRC. CUR suppressed the mRNA and protein expression of Nrf2, which further reduced the expression levels of Bcl-2. These effects promoted apoptosis in HCT-8/5-FU cells and reversed MDR.

OXA is a platinum-based chemotherapeutic agent that plays an important role in the treatment of CRC (Yin et al., 2019); nevertheless, the development of resistance limits its efficacy. Han et al. (2020) established the OXA-resistant CRC cell line HCT116/OXA, and found that CUR could reverse resistance to its OXA. Furthermore, they suggested that the mechanism underlying this effect may be the inhibition of excision repair cross-complementation 1 (ERCC1) through upregulation of miRNA-409-3p. These alterations result in inhibition of the expression of drug resistance-related proteins [Bcl-2, glutathione S-transferase-π (GST-π), mitochondrial ribosomal protein (MRP), P-gp, and survivin]. Yin et al. (2019) also established an OXA-resistant cell line (HCT116/OXA) to investigate the effect of CUR on resistance to OXA in CRC. According to the results, CUR could reverse resistance to OXA in CRC through inhibition of EMT by suppressing the transforming growth factor-β/Smads (TGF-β/Smads) pathway both *in vitro* and *in vivo*. TGF-β, regulated by Smad and non-Smad signaling pathways, is an important inducer of EMT (Katsuno et al., 2013). EMT has been gradually recognized as an important mechanism in the development of drug resistance in tumors (Yin et al., 2019). Ruiz et al. (Ruiz de Porras et al., 2016) found that NF-κB was overactivated in OXA-resistant CRC cells, and this overactivation could be attenuated by treatment with CUR. Meanwhile, they found that treatment with CUR plus OXA could revert resistance to OXA, potentially through inhibition of the NF-κB signaling cascade. In addition, the investigators reported that C-X-C motif chemokine ligand 1 (CXCL1) was regulated by NF-κB; its expression was upregulated in resistant cells, but downregulated after treatment with CUR plus OXA. Thus, they speculated that CXCL1 could be used as a predictive marker in patients with CRC. The majority of studies on CRC cells are conducted *in vitro*. Guo et al. (Guo L.-d. et al, 2015) evaluated the therapeutic effect of CUR and OXA *in vivo* by establishing xenografts through subcutaneous implantation of LoVo CRC cells in nude mice. The results showed that the combinatorial administration of CUR and OXA inhibited the growth of CRC in nude mice, caused cell cycle arrest, and induced apoptosis. In addition, some important signaling molecules were tested; the results showed that the expression of Bax, CASP3, and PARP was significantly increased, whereas that of Bcl-2, survivin, HSP70, pro-CASP3, and pro-PARP was markedly suppressed in tumor cells.

Su et al. (2018) also reported that CUR could reduce resistance to irinotecan (CPT-11). LoVo/CPT-11, an established CPT-11-resistant CRC cell line, exhibited markedly higher expression levels (mRNA and protein) of colon CSC markers (CD44, CD133, epithelial cell adhesion molecule [EpCAM], and CD24) compared with parental cells. Furthermore, treatment with CUR attenuated resistance to CPT-11, accompanied by reduction in the expression levels of CSC identification markers. In addition, they found that CUR induced cell apoptosis in sphere-forming cells, and the combination of CUR and CPT-11 could increase the apoptosis-inducing effect.

FOLFOX is the most commonly used combination chemotherapeutic regimen for CRC (Al Sabbagh et al., 2021). In recent years, studies (including clinical trials) on CUR and FOLFOX have been conducted. James et al. (James et al., 2015) established *ex vivo* CSC models from patient-derived colorectal liver metastases. They found that the combination of CUR and 5-FU/OXA could significantly reduce the number of spheroids, while the levels of CSC markers (ALDH and CD133) were also decreased. A phase I clinical trial (James et al., 2015) performed in the United Kingdom in patients with liver metastases from CRC showed that addition of daily oral CUR (0.5–2 g) to FOLFOX chemotherapy is safe and tolerable. A phase IIa (Howells et al., 2019), two-armed, randomized, and controlled clinical trial further demonstrated the safety and tolerability of this combination in patients with metastatic CRC who were randomly assigned to receive either FOLFOX or CUFOX (FOLFOX +2 g oral CUR per day). A significant difference in overall survival was observed between patients receiving FOLFOX and CUFOX. The concentration of plasma CXCL1 [growth-regulated oncogene-α (GRO-α)], which is associated with metastatic spread in CRC, was 1.7-fold higher in the FOLFOX group versus the CUFOX group; however, the difference was not statistically significant. Hence, it is suggested that the combination treatment has the potential to provide benefit to patients, though the currently available evidence is insufficient. Both phase I and II trials involved a small number of patients (12 and 28, respectively) and lacked a placebo control group. Thus, the clinical efficacy of CUR as an adjunct of FOLFOX chemotherapy is uncertain, and phase III trials are warranted.

## 3 Curcumin in gastric cancer

**FIGURE 2:**
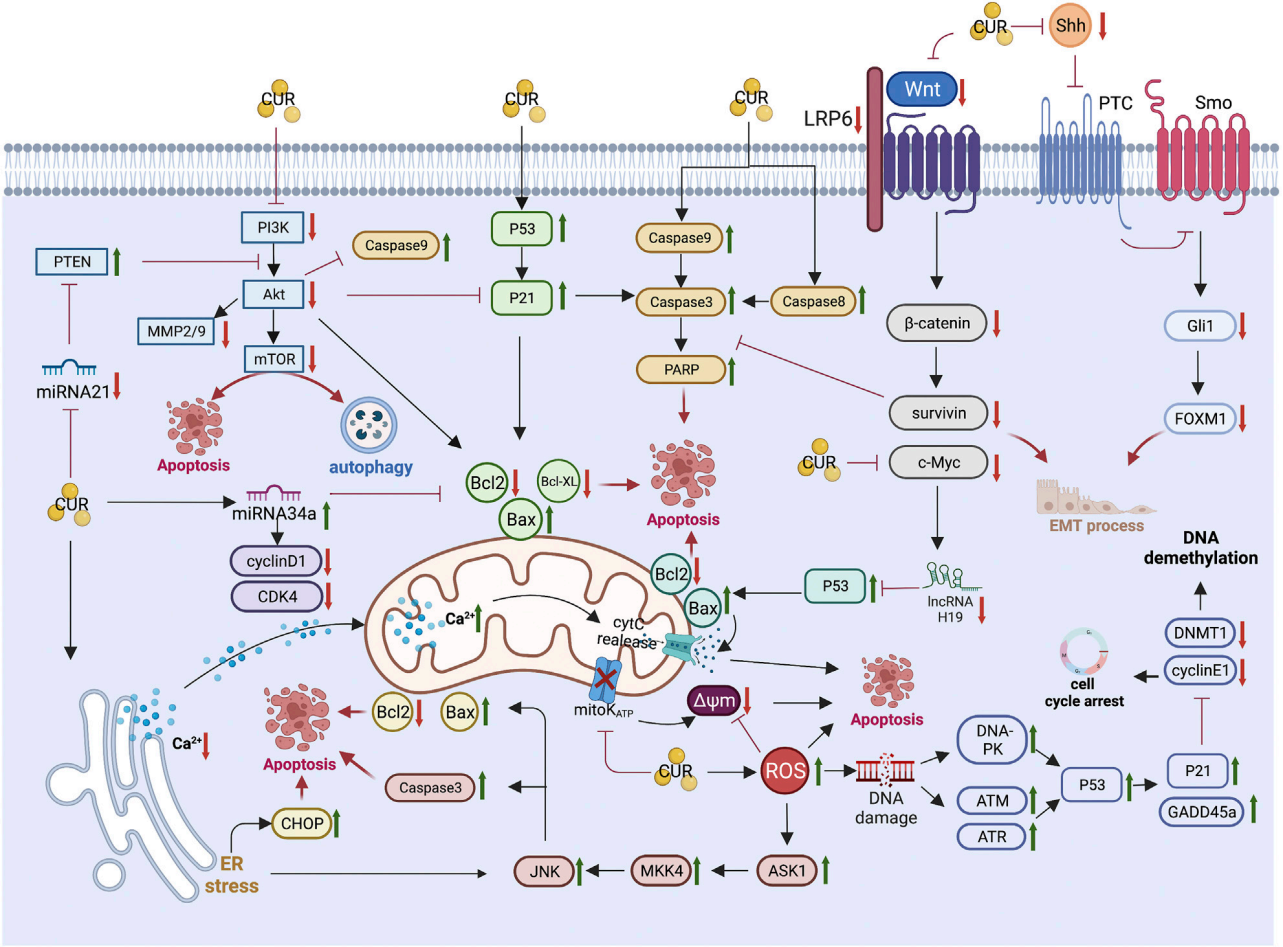
Pharmacological mechanism underlying the effects of curcumin on gastric cancer (Created with Biorender.com). PI3K, Phosphatidylinositol 3-kinase; Akt, Protein kinase B; mTOR, mammalian target of rapamycin; MMP2/9, matrix metalloproteinase 2/9; PTEN, phosphatase and tensin homolog; CDK4, cyclin-dependent kinase 4; Bcl2,B-cell lymphoma 2; Bax, B-cell lymphoma-2 (Bcl-2)-associated X protein; PARP, Poly (ADP-ribose) polymerase; LRP6, lipoprotein receptor-related protein 6; Shh, sonic hedgehog; Gli1, Glioma-associated oncogene homologue 1; FoxM1, forkhead box transcription factor M1; EMT, epithelial-mesenchymal transition; cytC, cytochrome C; Δψm, mitochondrial membrane potential; ER, endoplasmic reticulum; CHOP, CCAAT/enhancer binding protein homologous protein; JNK, c-Jun N-terminal kinases; MKK4, mitogen-activated protein kinase kinase 4; ASK1, apoptosis signal-regulated kinase 1; ROS, reactive oxygen species; DNA-PK, DNA-dependent protein kinase; ATM, Ataxia-telangiectasia mutated; ATR, ataxia telangiectasia and Rad3-related; GADD45a, growth arrest and DNA damage-inducible protein GADD45 alpha; DNMT1, DNA methyltransferase 1.

The unique anti-GC mechanism of CUR, which is described in detail below, combined with the above common anti-GIC mechanism of CUR is illustrated in Figure 2 and summarized in Table 1.

**TABLE 1:** Pharmacological mechanism underlying the effects of curcumin on gastric cancer.

Model(s)	Cell line(s)	Dose(s)	Mechanism(s)	References
*In vitro*	SGC7901	50 μM	Blockage of cell cycle	Sun et al. (2019)
*In vivo*			↑ miR-34a	
			↓ Bcl-2, CDK4, and CCND1	
*In vitro*	SGC7901	0, 10, 20, 40 μM	Induction of apoptosis and autophagy	Fu et al. (2018)
	BGC823		Activation of the p53 signaling pathway	
			Inhibition of the PI3K/Akt/mTOR signaling pathway	
*In vitro*	SGC7901	0–200 μM	Induction of apoptosis and autophagy	Li et al. (2017a)
	BGC823		Inhibition of the PI3K/Akt/mTOR signaling pathway	
	MKN28			
*In vitro*	AGS	30 μmol/L	Induction of apoptosis	Liu et al. (2018)
			Inhibition of migration and invasion	
			Inhibition of the PI3K/Akt signaling pathway	
			↓ miRNA-21	
*In vitro*	SNU-1	Cells: 8, 16, 32 μM	Induction of apoptosis	Zheng et al. (2017)
*In vivo*	SNU-5	Animals: 1 mg/kg bw per day	Inhibition of the Wnt/β-catenin signaling pathway	
	AGS			
*In vitro*	SGC7901	30 μM	Induction of apoptosis	Zhang et al. (2020)
			Inhibition of migration, invasion, EMT, and cytoskeletal remodeling	
			Inhibition of the Wnt/β-catenin and GLI1-β-catenin pathway	
*In vitro*	MGC803	15 μmol/L	Induction of apoptosis	He et al. (2017)
			Activation of the CASP3 pathway	
			↓ Bcl-2	
			↑ Bax	
*In vitro*	MGC803	5–40 μM	Induction of apoptosis	Qiang et al. (2019)
			Inhibition of the miR-21/PTEN/Akt pathway	
*In vitro*	SGC7901	5, 10, 25, 50 μmol/L	↓ H19	Liu et al. (2016)
			Inhibition of the c-Myc/H19 pathway	
*In vitro*	SGC7901	Cells: 50 μM	↑ miR-34a	Sun et al. (2019)
*In vivo*			↓ Bcl-2	
			↓ CDK4 and CCND1	
*In vitro*	hGCC	10, 15, 20, 40, 60 μM	↑ ROS	Tong et al. (2020)
			Induction of mitochondrial damage, DNA damage, and apoptosis	
*In vitro*	BGC-823	5, 10, 15, 20, 40 μM	Induction of apoptosis	Liang et al. (2014)
			↑ ROS	
			Regulation of the ASK1-MKK4-JNK pathway	
*In vitro*	AGS	2.5–80 μM	Induction of apoptosis	Cao et al. (2013)
			Induction of ER stress and mitochondrial dysfunction	
*In vivo*	MFC	20, 40, 60 μmol/L per day, intragastrically	Induction of apoptosis	Wang et al. (2017)
			↓ DEC1, HIF1A, VEGF, and STAT3 pathways	
*In vitro*	GC-MSC	30 μM	Inhibition of angiogenesis	Huang et al. (2017)
	HGC-27		Inhibition of the NF-κB/VEGF pathway	
	HUVECs			
*In vivo*	SGC-7901	40, 80, 160 mg/kg bw per day	Inhibition of gastric cancer lymph node metastasis	Da et al. (2015)
*In vitro*	AGS	CUR: 25 μg/ml	Enhancement of the chemotherapeutic effect of DOX	Amoodizaj et al. (2020)
		DOX: 15 μg/ml		
		DOX-CUR: 5 μg/ml		
*In vitro*	SGC7901	CUR: 40 μmol/L	Reversal of chemoresistance	Yu et al. (2011)
		DOX: 0.3 μmol/L	↓ NF-κB	
		Etoposide: 20 μmol/L		
*In vitro*	SGC7901	5- FU: 10 μM	Reversal of resistance to 5-FU	Kang et al. (2016)
		CUR: 20 μM	Inhibition of the NF-κB pathway	
*In vitro*	MGC803	15 μmol/L	Enhancement of the effects of treatment with FP	He et al. (2017)
*In vitro*	BGC-823	*In vitro*: 10 μM	Enhancement of the effects of treatment with FOLFOX	Zhou et al. (2016)
*In vivo*		*In vivo*: 10 mg/kg bw per day, intraperitoneally		
*In vitro*	SGC7901	*In vitro*: 25 μM	Suppression of gastrin-mediated acid secretion	Zhou et al. (2017)
*In vivo*		*In vivo*: 100 mg/kg bw, *per os*		

5-FU, 5-fluorouracil; ASK1-MKK4-JNK, apoptosis signal regulating kinase 1-mitogen-activated protein kinase 4-JUN N-terminal kinase; Bax, Bcl-2-associated X; Bcl-2, B-cell lymphoma 2; bw, body weight; CASP3, caspase 3; CCND1, cyclin D1; CDK4, cyclin-dependent kinase 4; CUR, curcumin; DEC1, differentiated embryonic chondrocyte-expressed gene 1; DOX, doxorubicin; EMT, epithelial–mesenchymal transition; ER, endoplasmic reticulum; FP, 5-FU, plus cisplatin; GC-MSC, gastric cancer cell-derived mesenchymal stem cells; GLI1, GLI, family zinc finger 1; hGCC, human gastric cancer cells; HER2, human epidermal growth factor receptor 2; HIF1A, hypoxia inducible factor 1 subunit alpha; HUVEC, human umbilical vein endothelial cells; mTOR: mechanistic target of rapamycin kinase; NF-κB, nuclear factor-κB; PAK1, p21-activated kinase 1; PI3K, phosphoinositide-3-kinase; PTEN, phosphatase and tensin homolog; ROS, reactive oxygen species; STAT3, signal transduction and transcription activator 3; VCR, vincristine; VEGF, vascular endothelial growth factor; ↑ denotes upregulation; ↓ denotes downregulation.

### 3.1 Activation of caspase 3 signaling pathway and regulation of Bcl-2-associated X, Bcl-2

Activation of the cysteine protease (caspase) family plays a critical role in apoptosis and is considered a direct effector of apoptosis. Caspase 3 (CASP3) is the main effector molecule of apoptosis; it can lyse DNA repair-related molecules, apoptosis inhibitory proteins, extracellular matrix proteins, and skeleton proteins. The activation of CASP3 signals an reliable marker for apoptosis (Crowley & Waterhouse, 2016). Members of the Bcl-2 family play a central role in the regulation of apoptosis and influence the cell cycle, particularly the transition from quiescence to proliferation (Xu et al., 2011). He et al. (He et al., 2017) demonstrated that CUR inhibited the proliferation and promoted apoptosis of MGC803 cells *via* the activation of CASP3/8, downregulation of Bcl-2, and upregulation of Bcl-2-associated X (Bax).

### 3.2 Downregulation of miRNA-21

It has been found that miRNA-21 is highly expressed in GC tissues (Liu et al., 2018). CUR induced the activity of phosphatase and tensin homolog (PTEN) and inhibited that of the PI3K/Akt signaling pathway by inhibiting miRNA-21 expression in AGS cells. These effects resulted in the inhibition of biological activity and induced apoptosis of AGS cells. Similar findings were also obtained in MGC803 cells (Liu et al., 2018). Qiang et al. (Qiang et al., 2019) found that CUR induced apoptosis, inhibited the expression of phospho-Akt protein, upregulated PTEN expression, and downregulated the levels of miRNA-21 in MGC803 cells. These results suggested that CUR negatively modulated the miR-21/PTEN/Akt pathway in MGC803 cells.

### 3.3 Downregulation of long non-coding RNA H19

It is gradually accepted that long non-coding RNA (lncRNAs) play multiple roles in the progression of cancer. Previous studies (Song et al., 2013; Wang J. et al, 2015) have reported that H19 is abnormally upregulated in GC. This upregulation directly inhibits p53 activation, thereby promoting GC progression (Yang et al., 2012). Liu et al. (Liu et al., 2016) found that CUR inhibited the expression of H19 and upregulated that of p53 in a concentration-dependent manner in SGC7901 cells. Further investigation showed that CUR downregulated the expression of the c-Myc oncogene. In addition, the downregulation of H19 expression induced by CUR could be reversed by exogenous c-Myc protein. This observation suggested that CUR inhibited the proliferation and induced apoptosis of GC cells by downregulating the c-Myc/H19 pathway.

### 3.4 Upregulation of miR-34a

It has been revealed that miR-34a plays a critical role in GC (Wong et al., 2011). Sun et al. (Sun et al., 2019) found that CUR could upregulate miR-34a expression, inhibit proliferation, and induce apoptosis in SGC7901 cells. Administration of CUR or miR-34a in GC cells or tissues decreased the expression of Bcl-2, CDK4, and CCND1. It is suggested that CUR may downregulate Bcl-2, CDK4, and CCND1 expression by upregulating miR-34a, thus inducing apoptosis in GC.

### 3.5 Activation of the endoplasmic reticulum stress pathway

Cao et al. (2013) indicated that CUR could function through endoplasmic reticulum (ER) stress and mitochondria functional pathways in GC cells AGS, based on the upregulation of CCAAT/enhancer binding protein homologous protein (CHOP), phosphorylation of JNK and downregulation of SERCA2ATPase, release of cytochrome c, reduction of Bcl-2, and loss of MMP.

### 3.6 Suppression of gastric acid secretion

The development of GC is often accompanied by excessive gastric acid secretion (Deng et al., 2012). It is widely thought that the acidic environment in the stomach contributes to the development of GC (Sheng et al., 2018). Therefore, hyperacidity may be related to the progression of GC. Gastrin, an inducer of gastric acid secretion, is a valuable marker for GC screening (Waldum et al., 2017). Zhou et al. (2017) evaluated the mechanism through which CUR inhibits GC by affecting gastric acid secretion both *in vitro* and *in vivo*. The results showed that CUR significantly inhibited GC progression through an increase in gastric pH and suppression of gastrin-mediated acid secretion.

### 3.7 Inhibition of angiogenesis and lymphangiogenesis

Vascular endothelial growth factor (VEGF) can promote angiogenesis and growth of cancer cells, and is considered the most important and specific angiogenic factor. Signal transduction and transcription activator 3 (STAT3) is highly expressed in GC, and is closely related to cancer cell stage, invasion depth, lymph node metastasis, and cancer grade. Overexpression of STAT3 and VEGF in GC cells induced an increase in microvascular density and disease progression. Using BALB/C mice grafted with a mouse gastric adenocarcinoma cell line MFC as an experimental model, Wang et al. (2017) investigated the antitumor activity of CUR. The results showed that CUR inhibited the proliferation of GC by downregulating the differentiated embryonic chondrocyte-expressed gene 1-hypoxia inducible factor 1 subunit alpha-STAT3-VEGF (DEC1-HIF1A-STAT3-VEGF) signal transduction pathway.

Huang et al. (2017) reported that tumor-derived mesenchymal stem cells (MSC) are important for tumor angiogenesis, and investigated the mechanism through which CUR mediates angiogenesis to regulate GC cell-derived MSC (GC-MSC). The results showed that CUR reduced GC-MSC-derived angiogenesis by inhibiting the NF-κB/VEGF signaling pathway, which plays a critical role in tube formation, migration, and colony formation induced by GC-MSC.

High mobility group box 1 (HMGB1) and vascular endothelial growth factor D (VEGFD) are associated with lymphangiogenesis and tumor metastasis in GC. Da et al. (2019) found that CUR may inhibit lymphangiogenesis in GC cells (AGS and SGC-7901) through the inhibition of HMGB1/VEGFD signaling. In addition, they investigated the effect of CUR on lymphatic vessel density in a nude mouse model using SGC7901 cells. The results indicated that CUR inhibited lymph node metastasis of GC, as evidenced by the reduced tumor volume, decreased protein expression of prospero homeobox 1 (PROX1), podoplanin (PDPN), and vascular endothelial growth factor receptor 3 (VEGFR3), and downregulated mRNA expression of lymphatic vessel endothelial receptor 1 (LYVE1), PROX1, PDPN, and VEGFR3 (Da et al., 2015).

## 4 Curcumin in colorectal cancer

**FIGURE 3:**
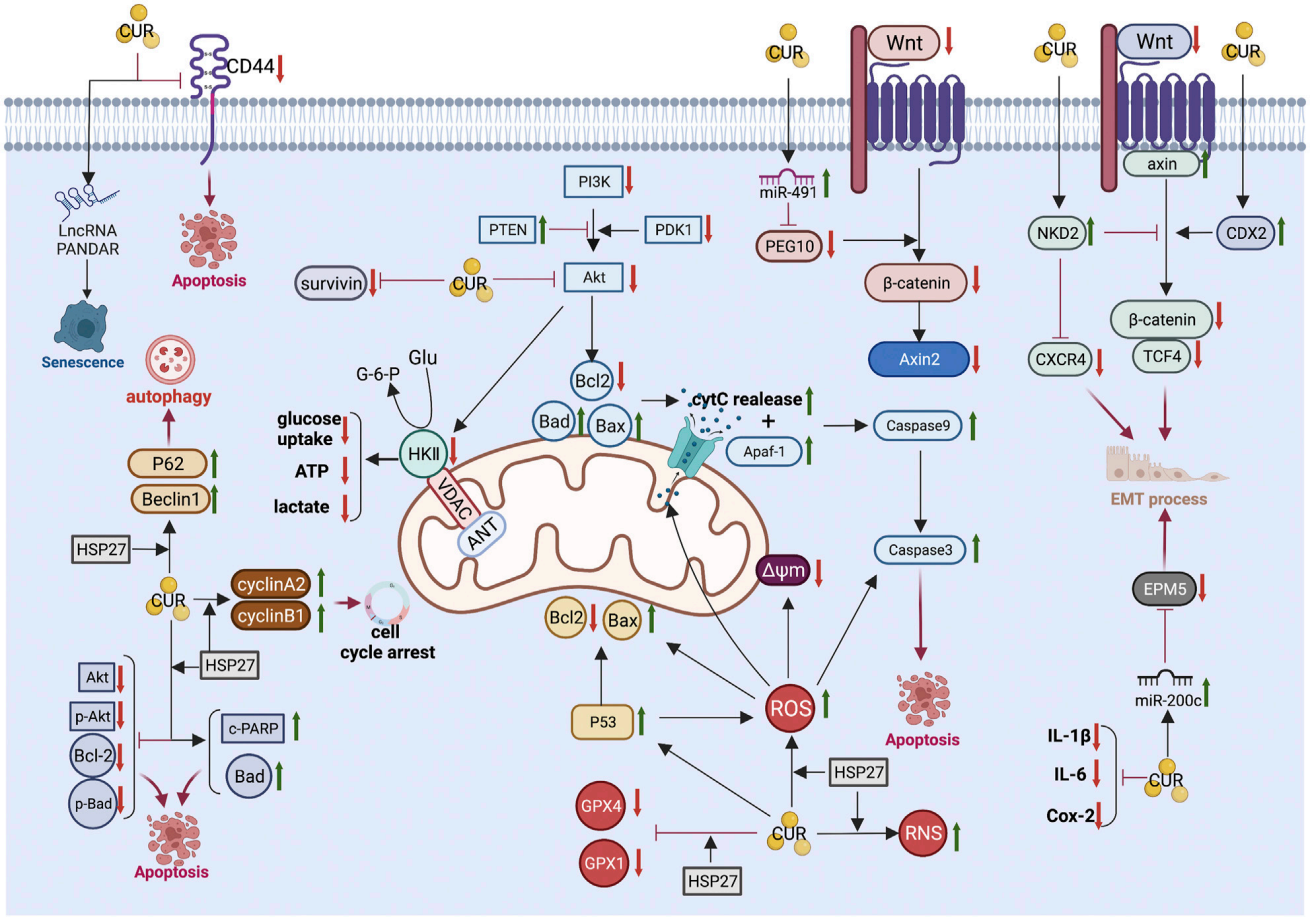
Pharmacological mechanism underlying the effects of curcumin on colorectal cancer (Created with Biorender.com). LncRNA, long non-coding RNA; PI3K, Phosphatidylinositol 3-kinase; Akt, Protein kinase B; PTEN, phosphatase and tensin homolog; PDK1, phosphoinositide-dependent protein kinase-1; Bcl2, B-cell lymphoma 2; Bax, B-cell lymphoma-2 (Bcl-2)-associated X protein; Bad, BCL2 associated agonist of cell death; cytC, cytochrome C; Δψm, mitochondrial membrane potential; Apaf-1, Apoptotic protease activating factor 1; PEG10, paternally expressed gene 10; Axin2, axis inhibition protein 2; NKD2, Naked cuticle homolog 2; CXCR4, chemokine receptor 4; CDX2, caudal type homeobox 2; TCF4, transcription factor 4; EMT, epithelial-mesenchymal transition; IL-6, interleukin-6; IL-1β, interleukin-1β; Cox-2,cyclooxygenase-2; ROS, reactive oxygen species; RNS, reactive nitrogen species; HSP27, Heat shock protein 27; GPX4, Glutathione peroxidase 4; GPX1, Glutathione peroxidase-1; c-PARP, cleaved poly (ADP-ribose) polymerase; GluGlucose.

The anti-CRC mechanism of CUR, which is described in detail below, combined with the above common anti-GIC mechanism of CUR is illustrated in Figure 3 and summarized in Table 2.

**TABLE 2:** Pharmacological mechanism underlying the effects of curcumin on colorectal cancer.

Model(s)	Cell line(s)	Dose(s)	Mechanism(s)	References
*In vitro*	CC531	*In vitro*: 15, 20, 25, 30 μM	Inhibition of cell proliferation	De La Parte et al. (2021)
*In vivo*		*In vivo*: 200 mg/kg bw per day, *per os*	Reduction of tumor volume in liver implants	
*In vitro*	CSCs	1, 5, 25 μM	Inhibition of tumorsphere formation	Mao et al. (2021)
			Decrease in cell viability	
			Induction of apoptosis and autophagy	
			Inhibition of the ECM pathway	
*In vitro*	Caco-2	5–10 μM	Blockage of the cell cycle	Blakemore et al. (2013)
	DLD-1			
	HCA-7, HCT116p53+/+			
	HCT116p53−/−			
	HCT116p21−/−			
	HT-29			
	SW480			
*In vivo*	1,2-dimethylhydrazine dihydrochloride induced colorectal carcinogenesis in rats	50 mg/kg bw per day, *per os*	↓ PI3K/Akt pathway	Rana et al. (2015)
			Activation of mitochondrial apoptosis	
*In vitro*	HCT116	12.5 μM	↑ miR-491	Li et al. (2018)
			↓ PEG10	
			Inhibition of the Wnt/β-catenin pathway	
			Induction of apoptosis	
*In vivo*	AOM-DSS induced CRC in mice	500 mg/kg bw per day, *per os*	↓ Axin2	Hao et al. (2021)
			Inhibition of the Wnt/β-catenin signaling pathway	
*In vitro*	HCT-116	5, 10, 20, 30 μM	↓ CD44	Fan et al. (2020)
	HCT-8			
*In vitro*	HCT116	10, 20, 40 μM	↓ HK2	Wang et al. (2015b)
			↓ Glycolysis	
	HT29		Induction of mitochondria-mediated apoptosis	
*In vitro*	LoVo	2.5, 5, 10, 20, 30 μg/ml	Induction of mitochondria-mediated apoptosis	Guo et al. (2013)
*In vivo*		360 mg thrice per day	↑ p53 and TNF-α	He et al. (2011)
			Induction of apoptosis	
*In vitro*	DLD-1	15, 20, 25 μM	Induction of apoptosis and autophagy	Liang et al. (2018)
	HT-29		Regulation of HSP27	
			↑ ROS	
*In vitro*	HT29	40 μM	Induction of apoptosis	Sritharan & Sivalingam, (2021)
	HCT116		↑ ROS	
*In vitro*	DLD-1	1.25, 2.5, 5 μM	↑ PANDAR	Chen et al. (2017)
			Induction of senescence and apoptosis	
*In vitro*	HCT-116	*In vitro*: 1, 10 μM	Binding to SIRT1 and reduction of its stability	Lee et al. (2018)
*In vivo*	DLD-1	*In vivo*: 50 mg/kg bw, 100 mg/kg bw, 8 times during 3 weeks		
	HCT-15			
*In vitro*	HT29	1–25 μM	Inhibition of anchorage-independent growth	Guo et al. (2015b)
			↑ DLEC1	
			↓ CpG methylation	
*In vitro*	SW620	10, 20, 40 μmol/L	Inhibition of EMT	Zhang et al. (2016)
			↑ NKD2	
			Inhibited Wnt/β-catenin pathway	
			↑ CXCR4	
*In vitro*	SW480	0, 0.1, 0.2, 0.4 μmol/L	Inhibition of EMT	Chen et al. (2020)
			↓ CDX2 promoter methylation	
			↑ CDX2	
			Inhibition of the Wnt/β-catenin pathway	
*In vitro*	SW620		Inhibition of EMT	Wang et al. (2020)
			↑ miR-200c	
	HT29		↓ EPM5	
*In vivo*	AOM-induced colon cancer model in IL-10 -/-mice	8–162 mg/kg bw per day	Modulation of colonic microbial ecology	McFadden et al. (2015)
	HCT-116	*In vitro*: 0–20 μM		Chen et al. (2013)
	HT-29			
	HCT-15		Suppression of metastasis	
*In vitro*	HCC-2998		↓ Sp-1, FAK, and CD24	
*In vivo*	Colo205	*In vivo*: 1 g/kg bw per day for 30 days	↑ E-cadherin	
	Km-12			
	SW-620			
*In vitro*	HCT116	0–75 μM	Inhibition of angiogenesis and metastasis	Calibasi-Kocal et al. (2019)
	LoVo			
*In vitro*	CC531	*In vitro*: 15, 20, 25, or 30 µM	Inhibition of migration, antitumor effect against liver	De La Parte et al. (2021)
*In vivo*	WAG/RijHsd tumor -bearing rats	*In vivo*: 200 mg/kg bw per day, *per os*	implants from CRC	
*In vitro*	HT-29	0–60 μM	Synergy with cisplatin to suppress the proliferation of CRC	Fan et al. (2022)
	LoVo		↑ miR-137	
	DLD-1		↓ Glutaminase	
	HT-116			
	SW480			
*In vitro*	HCT8	*In vitro*: 0,1,3,10 μM	Reversal of resistance to cisplatin	Zheng et al. (2021b)
*In vivo*	HCT8/DDP	*In vivo*: 1 g/kg bw weekly, intraperitoneal injection	↓ KCNQ1OT1 and Bcl-2	
			↑ miR-497	
*In vitro*	SW480	*In vitro*: 0–60 μM	Reversal of resistance to 5-FU	Li et al. (2021a)
*In vivo*	HT-29	*In vivo*: 100 mg/kg, intragastrically, once every 2 days	↓ NNMT	
			↑ ROS, G2/M phase cell cycle arrest	
*In vitro*	SW620	*In vitro*: 10 μmol/L CUR; 5 μmol/L 5-FU	Enhancement of the anticancer effect of 5-FU	Zheng et al. (2021a)
*In vivo*	SW620 xenograft mice model	*In vivo*: intratumorally	↓ STAT1 and L1	
			Induction of apoptosis	
*In vitro*	HCT-116	5, 10, 20, 40 μM	Reversal of resistance to 5-FU	Lu et al. (2020)
			↓ TET1 and NKD2	
			Inhibition of the WNT signaling pathway and EMT	
*In vitro*	HCT-8/5-FU	10 µM CUR	Reversal of resistance to 5-FU	Zhang et al. (2018)
		10 mM 5-FU	↓ Nrf2 and Bcl-2	
			Induction of apoptosis	
*In vitro*	HCT116/OXA	0–40 μM	Reversal of resistance to OXA	Han et al. (2020)
			↑ miR-409-3p	
			↓ ERCC1, Bcl-2, GST-π, MRP, P-gp, and survivin	
*In vitro*	HCT116/OXA	*In vitro*: 8 μM CUR; 4 μM OXA	Reversal of resistance to OXA	Yin et al. (2019)
*In vivo*		*In vivo*: 60 mg/kg bw per day, intraperitoneal injection	Inhibition of EMT and TGF-β/Smads signaling	
*In vitro*	HT29	1.25–30 μM	Reversal of resistance to OXA	Ruiz de Porras et al. (2016)
	LoVo		Inhibition of the CXC-chemokine/NF-κB pathway	
	DLD-1			
*In vivo*	Xenografts with LoVo cells	CUR: 50 mg/kg bw	Enhancement of the anticancer effect of OXA	Guo et al. (2015a)
			Induction of apoptosis, cell cycle arrest	
		OXA: 25 mg/kg bw, intraperitoneal injection, thrice weekly, for 22 days	↑ Bax	
			↓ Bcl-2, survivin, HSP70, c-Myc, pro-CASP3, pro-PARP, CASP3, PARP	
*In vitro*	LoVo/CPT-11	0–20 µM	Attenuation of resistance to irinotecan	Su et al. (2018)
			Induction of apoptosis in CSC	
*Ex vivo* and *in vivo* (human)	CRLM CSC model, human (phase I trial)	CRLM model: 5 μM	Reduction of the spheroid number and CSC markers	James et al. (2015)
		Patients: 0.5, 1, 2 g, daily, *per os*	Safe and tolerable to patients	
*In vivo* (human)	Human (phase IIa trial)	Patients: 2 g daily, *per os*	Safe and tolerable to patients	Howells et al. (2019)

5-FU, 5-fluorouracil; AOM, azoxymethane; Bax, Bcl-2-associated X; Bcl-2, B-cell lymphoma 2; bw, body weight; CASP3, caspase 3; CDX2, caudal type homeobox 2; CPT-11, irinotecan; CRC, colorectal cancer; CRLM, colorectal liver metastasis; CSC, cancer stem cell; CUR, curcumin; CXCR4, C-X-C motif chemokine receptor 4; DLEC1, deleted in lung and esophageal cancer 1; DSS, dextran sulfate sodium; ECM, extracellular matrix; EMT, epithelial–mesenchymal transition; GST-π, glutathione S-transferase-π; HK2, hexokinase 2; HSP27, heat shock protein 27; IL-10, interleukin-10; KCNQ1OT1, KCNQ1 opposite strand/antisense transcript 1; MRP, mitochondrial ribosomal protein; NF-κB, nuclear factor-κB; NKD2, naked cuticle homolog 2; NNMT, nicotinamide N-methyltransferase; Nrf2, nuclear factor erythroid 2-related factor; OXA, oxaliplatin; PANDAR, promoter of CDKN1A antisense DNA, damage activated RNA; PARP, poly(ADP-ribose) polymerase; PEG10, paternally expressed gene 10; P-gp, P-glycoprotein; PI3K, phosphoinositide-3-kinase; ROS, reactive oxygen species; STAT1, signal transduction and transcription activator 1; SIRT1, sirtuin 1; TGF-β, transforming growth factor-β; TNF-α, tumor necrosis factor-α; ↑ denotes upregulation; ↓ denotes downregulation.

### 4.1 Downregulation of CD44 expression

CD44, a family of transmembrane glycoproteins, is an important cancer stem cell marker in CRC. It is involved in multiple cellular biological functions, such as proliferation, migration, and survival (Thapa & Wilson, 2016; Chen et al., 2018). Fan et al. (Fan et al., 2020) examined the relationship between the anticancer effect of CUR and CD44 in CRC. Following treatment with CUR, the expression of CD44 was downregulated in HCT-116 and HCT-8 cell lines in a dose-dependent manner. In addition, CUR specifically inhibited CD44 ^+^ CRC cells. Knockdown of CD44 expression using siRNA led to a significant decrease in the inhibitory effect of CUR against CD44 ^+^ CRC cells. Further investigation indicated that the apoptosis of CD44 ^+^ CRC cells induced by CUR may be associated with the binding of CUR and the CD44 molecule, which could result in a higher uptake of CUR.

### 4.2 Regulation of heat shock protein 27

Heat shock protein 27 (HSP27), a small heat shock protein, was found to be lowly expressed in normal cells; however, its levels increase following exposure to survival stress (Hou et al., 2016). High expression of HSP27 has been associated with growth, progression, and metastasis of cancer cells (Hung et al., 2017).

Liang et al. (2018) demonstrated that silencing of HSP27 inhibited apoptosis and autophagy, consequently inducing resistance to treatment with CUR in CRC cells. They found that DLD-1 cells (with high HSP27 expression) are more sensitive to treatment with CUR than HT-29 cells (with low HSP27 expression). HSP27 knockdown (HSP27-KD) cells presented resistance to CUR, accompanied by decreased levels of antiapoptotic proteins (phospho-Akt, Akt, Bcl-2, and phospho-Bad) and increased levels of apoptotic proteins (Bad and cleaved PARP). A series of CUR-induced effects were abolished in HSP27-KD cells, including apoptosis, G2/M cell cycle arrest, oxidative stress, autophagy, blockage of Akt signaling, and mitochondria-mediated apoptosis. In addition, HSP27-KD cells had decreased levels of antiapoptotic proteins (phospho-Akt, Akt, Bcl-2, and phospho-Bad) and increased levels of apoptotic proteins (Bad and cleaved PARP). These data indicated that HSP27-KD induced resistance to CUR in CRC cells.

### 4.3 Induction of senescence

Chen et al. (2017) demonstrated that treatment with low-dose CUR induced senescence and promoted CDKN1A antisense DNA damage activated RNA (PANDAR) expression in CRC DLD-1 cells. Notably, silencing of lncRNA PANDAR in these cells may switch senescence to apoptosis partly by regulating the expression of p53-upregulated modulator of apoptosis (PUMA). They did not find a difference in the expression of PANDAR between CRC tissues and corresponding normal tissues. Knockdown of PANDAR by si-RNA did not significantly change the proliferation of CRC DLD-1 cells. Moreover, low-dose CUR (5 μM) induced senescence but not apoptosis in DLD-1 cells, along with upregulation of PANDAR expression. Interestingly, silencing of PANDAR in DLD-1 cells treated with CUR was linked to enhanced apoptosis and a significant reduction in senescent cells. They also found that silencing of PANDAR stimulated the mRNA expression of the pro-apoptotic gene PUMA in DLD-1 cells treated with CUR.

### 4.4 Binding to sirtuin 1 and reduction of its stability

Sirtuin 1 (SIRT1) is a NAD+-dependent histone/protein deacetylase with several physiological functions, such as metabolic regulation, differentiation, and stress response (Lee et al., 2018). It is involved in the progression of CRC (Song et al., 2018) and highly expressed in human CRC tissues compared with adjacent normal tissues (Song & Surh, 2012). Lee et al. (Lee et al., 2018) found that CUR downregulated the expression of SIRT1 protein, but not mRNA, in human CRC cells (HCT-116, DLD-1, and HCT-15). These findings suggested that the regulation of SIRT1 by treatment with CUR is post-translational. Results obtained through nano-liquid chromatography tandem mass spectrometry indicated that CUR directly bound to cysteine 67 of SIRT1 and resulted in structural modification. Consequently, it promoted the ubiquitin-dependent proteasomal degradation of SIRT1, which is overexpressed in CRC cells. In conclusion, by binding to SIRT1 and decreasing its stability, CUR suppressed its oncogenicity in human CRC cells and inhibited disease progression both *in vitro* and *in vivo.*

### 4.5 Inhibition of anchorage-independent growth of colorectal cancer cells

As an *in vitro* characteristic of tumorigenic cells, anchorage-independent growth (colony-forming capacity in semisolid medium) has been considered a marker of transformed cells which differentiates them from normal cells (Borowicz et al., 2014). In tumor cells, the anchorage-independent growth is related to their tumorigenic and metastatic potential *in vivo* (Choi et al., 2021). The aberrant epigenetic landscape (i.e., heritable alterations in gene expression without changes in DNA sequence) may increase the complexity of CRC initiation and progression.

Deleted in lung and esophageal cancer 1 (DLEC1), a tumor suppressor gene, could reduce transcriptional activity and promote hypermethylation in several types of cancer, including CRC (Guo Y. et al, 2015). Guo et al. (Guo Y. et al, 2015) investigated the inhibitory role of DLEC1 in anchorage-independent growth of human colorectal adenocarcinoma HT29 cells and epigenetic regulation by CUR. The results revealed the tumor inhibitory role of DLEC1, and indicated its association with the inhibition of anchorage-independent growth of HT29 cells by treatment with CUR. In addition, the investigators demonstrated that CUR suppressed the anchorage-independent growth of HT29 cells by upregulating DLEC1 and reducing CpG methylation in HT29 cells. This activity may be involved in lowering the protein expression of DNMTs and HDACs.

### 4.6 Modulation of colonic microbial ecology

It has been well established that the inflammatory microenvironment and intestinal microbiota influence the progression of colitis-associated CRC (Schwabe & Jobin, 2013; McFadden et al., 2015). McFadden et al. (McFadden et al., 2015) used interleukin-10 (IL-10) -/- mice on 129/SvEv background as a model of colitis-associated CRC to evaluate the role of CUR in modulating colonic microbial ecology and preventing the progression of chronic colitis to CRC. Wild-type or IL-10 -/- mice (age: 10 weeks) received six weekly intraperitoneal injections of azoxymethane or phosphate-buffered saline, along with supplementation with a control or CUR initiated at the same time. Stools were collected every 4 weeks for microbial community analysis. The results showed that dietary CUR reduced or entirely prevented the development of CRC in the azoxymethane-induced CRC model in a dose-dependent manner. The chemoprophylactic effects appeared to be related to the ecological regulation of colonic microorganisms by CUR, rather than the reduction of inflammation. These findings suggested that the role of CUR in CRC prevention is associated with the maintenance of a more diverse colon microbial ecology.

## 5 Conclusion

GIC are associated with high morbidity and mortality rates, thus posing a serious threat to human life and health. Currently, surgery and chemotherapy are the main treatment options for GIC; nevertheless, their efficacy is limited. In numerous studies, CUR, a plant extract with a good safety profile, has exhibited pharmacological effects on GIC both *in vivo* and *in vitro*. As demonstrated in the present review, CUR can effectively inhibit GlC through multiple targets, mechanisms, and pathways. Despite the potential of CUR in drug development and application to the treatment of GIC, there remain some deficiencies in the current studies that warrant further investigation.

Regulated cell death (RCD) mainly includes apoptosis, autophagy, pyroptosis, necroptosis, and ferroptosis. Studies investigating RCD induced by CUR in GIC mainly focus on apoptosis and autophagy. It remains unclear whether CUR could induce pyroptosis, necroptosis, and ferroptosis in GIC. These three modes of RCD were recently proposed, and have become hot topics in antitumor drug research, particularly with regard to apoptosis-resistant cells. It has been reported that CUR promoted pyroptosis in liver cancer cells (Li W.-f. et al, 2021), induced necroptosis in prostate and lung cancer cells (Lee et al., 2021), and induced ferroptosis in breast (Li et al., 2020) and lung cancer cells (Tang et al., 2021). Further research on the potential induction of pyroptosis, necroptosis, and ferroptosis by CUR in GIC, as well as the mechanisms involved in these processes, is required.

CUR has exhibited good antitumor activity and low toxicity. Nevertheless, its poor solubility in water (456 μg/L) and poor stability in aqueous solution result in an insufficient concentration in serum and tissues, leading to low bioavailability and clinical efficacy (Jakubek et al., 2019). To resolve this problem, researchers have turned their attention to CUR analogs and nanopreparations. This attempt is focused on overcoming its deficiencies in terms of water solubility, bioavailability, and stability, while retaining its excellent antitumor effect. CUR analogs (e.g., WZ35 (He et al., 2019), M37 (Liang et al., 2017), and EF24 (He et al., 2016)) and CUR nanoparticles (e.g., CUR micelles (Lin et al., 2020), CUR-carrying nanoliposomes (Angeline et al., 2020), and solid lipid nanoparticle (Mohamed et al., 2021)) have exerted excellent antitumor effects on GIC. This evidence suggests a breakthrough in the application of CUR to the treatment of GIC and provides broad research prospects.
